# 基于超高效液相色谱-紫外检测定量指纹图谱结合化学模式识别的复方金钱草颗粒质量评价

**DOI:** 10.3724/SP.J.1123.2022.07021

**Published:** 2022-12-08

**Authors:** Shaoming LU, Xin XU, Qianqian XUE, Liujun XIAO, Wenyi YU, Tong WEI, Hongli JIN, Yanfang LIU, Xinmiao LIANG

**Affiliations:** 1.赣江中药创新中心, 江西 南昌 330000; 1. Ganjiang Chinese Medicine Innovation Center, Nanchang 330000, China; 2.江西省中药药效物质基础重点实验室, 江西 南昌 330000; 2. Jiangxi Provincial Key Laboratory for Pharmacodynamic Material Basis of Traditional Chinese Medicine, Nanchang 330000, China; 3.中科院大化所中国医药城生物医药创新研究院, 江苏 泰州 225300; 3. DICP-CMC Innovation Institute of Medicine, Taizhou 225300, China; 4.中国科学院大连化学物理研究所, 辽宁 大连 116023; 4. Dalian Institute of Chemical Physics, Chinese Academy of Sciences, Dalian 116023, China

**Keywords:** 定量指纹图谱, 相似度分析, 聚类分析, 主成分分析, 正交偏最小二乘判别分析, 芒果苷, 异芒果苷, 复方金钱草颗粒, quantitative fingerprint, similarity analysis, hierarchical cluster analysis (HCA), principal component analysis (PCA), orthogonal partial least squares discriminant analysis (OPLS-DA), mangiferin, isomangiferin, Fufang Jinqiancao granules

## Abstract

复方金钱草颗粒具有利尿、抑制泌尿系结石形成、抗炎、抗氧化作用,且具有较大的市场需求。因此,采用超高效液相色谱-紫外检测(UPLC-UV)法建立定量指纹图谱,并结合化学模式识别技术对不同年份的复方金钱草颗粒进行质量评价,可为其质量控制提供依据。采用聚类分析(HCA)和主成分分析(PCA)等化学模式识别技术对35批复方金钱草颗粒样品的指纹图谱数据进行分析,筛选出质量差异标志物芒果苷和异芒果苷,并对二者进行含量测定。在复方金钱草颗粒指纹图谱中共指认出12个共有峰,且35批样品的相似度均在0.952以上。在HCA中,将35批样品分为了两类,其中2018年和2019年的样品为一类,2020年和2021年的样品为一类。此外,PCA结果显示了与聚类分析相同的聚类趋势。在此基础上,进一步通过正交偏最小二乘法分析 (OPLS-DA)筛选出了导致2018年、2019年与2020年、2021年的样品产生差异的差异标志物芒果苷和异芒果苷。以两个差异标志物芒果苷和异芒果苷为指标进行含量测定,结果显示色谱峰的分离度良好,线性关系良好,平均加标回收率分别为101.7%~105.6%和103.4%~105.5%,且相对标准偏差(RSD)均低于1.43%。在35批样品中,2020年、2021年的样品与2018年、2019年的样品相比,芒果苷与异芒果苷含量更高且波动范围更小。该研究建立了准确、可靠的复方金钱草颗粒质控方法,实现了对不同年份的复方金钱草颗粒样品合理、有效的质量评价,可为建立更系统、更全面的质量控制标准提供借鉴与参考。

## Abstract (en)

Fufang Jinqiancao granules have a large market demand due to the fact that they contain diuretics, inhibit urinary calculi formation, and exhibit both anti-inflammatory and antioxidant effects. In the current study, a fast and efficient quantitative ultra performance liquid chromatography-ultraviolet detection (UPLC-UV) fingerprinting method was established to analyze the Fufang Jinqiancao granules, while a chemical pattern recognition technology was used to evaluate the quality of the granules over different years. More specifically, the UPLC-UV system consisted of a Waters Acquity UPLC BEH C18 column (100 mm×2.1 mm, 1.7 μm), acetonitrile (mobile phase A), and a 0.1% formic acid aqueous solution (mobile phase B), wherein a gradient elution protocol was followed. Ultra performance liquid chromatography-quadrupole-time of flight-mass spectrometry (UPLC-Q-TOF-MS, Agilent Infinity Ⅱ 1290-6545) was used in combination with reference substances and literature comparisons to identify the common peaks present in the quantitative fingerprint. The fingerprints of 35 batches of Fufang Jinqiancao granules were established by means of the quantitative UPLC-UV fingerprinting method, and the fingerprint data obtained for these samples were further analyzed by chemical pattern recognition techniques, including hierarchical cluster analysis (HCA) and principal component analysis (PCA). The quality difference markers, namely mangiferin and isomangiferin, were screened, and their contents were determined. It was found that 12 common peaks existed in the fingerprint of the Fufang Jinqiancao granules, and the similarities of all 35 batches of samples were greater than 0.952. In addition, for the purpose of HCA, the 35 batches were divided into two categories, of which sample years 2018 and 2019 belonged to one category, and sample years 2020 and 2021 belonged to another category. Notably, PCA gave the same clustering trends as HCA. Based on the obtained results, the mangiferin and isomangiferin components responsible for the differences between the 2018, 2019 and 2020, 2021 samples were further screened by orthogonal partial least squares discriminant analysis (OPLS-DA). Moreover, the contents of the 35 batches of samples were determined using the two differential markers mangiferin and isomangiferin as indicators. The obtained results indicated that the chromatographic peaks of all 35 batches had acceptable resolutions, with mangiferin exhibiting a good linear relationship in the range of 5.3291-133.2276 mg/L, and isomangiferin exhibiting a similar linear relationship in the range of 4.1847-104.6170 mg/L. The average recovery of mangiferin was 101.7%-105.6%, with a relative standard deviation (RSD) of 0.63%-1.43%, while that of isomangiferin was 103.4%-105.5%, with an RSD of 0.60%-1.18%. Importantly, all RSD values were less than 1.43%, thereby indicating that our method meets the requirements of the Chinese Pharmacopoeia (2020 Edition). Among the 35 batches of samples, the contents of mangiferin and isomangiferin were higher in the 2020 and 2021 samples, and the content fluctuation range was smaller. Overall, the development of an accurate and reliable quality control method for Fufang Jinqiancao granules, and a reasonable and effective quality evaluation of Fufang Jinqiancao granule samples from different years was realized. We therefore expect that this study will provide a reference for establishing a more systematic and comprehensive quality control system.

复方金钱草颗粒是由广金钱草(GJQC)、车前草(CQC)、光石韦(GSW)、玉米须(YMX)4味中药材为主要原料加工制成的中药制剂,有清热利湿、通淋排石的功效^[1]^。现代药理学研究表明,复方金钱草颗粒具有利尿、抑制泌尿系结石形成、抗炎、抗氧化作用,能有效抑制结晶肾损伤,在治疗泌尿系结石、尿路感染等症时有显著功效^[2,3]^。复方金钱草颗粒已被列入2021年国家基本医疗保险用药目录。复方金钱草颗粒中含有黄酮类、多糖类、氨基酸类、挥发油类等多种有效成分^[4⇓⇓-7]^,但现行《中国药典》(2020版)仅对光石韦中的芒果苷进行了含量测定^[1]^。不同于化学药品成分的单一性,中药复方制剂具有多成分和多靶点的作用特点。使用单一指标成分的定性、定量分析方法并不能全面反映中药的内在质量^[8]^。因此,建立一种整体、全面的复方金钱草颗粒质量控制方法具有重要意义。本课题组2008年首次提出基于定量指纹图谱技术的中药质量控制策略,在原有指纹图谱的方法上赋予其大量的定性、定量信息,并将这些信息共同应用于中药的质量控制;通过对指纹图谱中的多个目标成分尤其是有效成分的准确定量描述可使中药质量控制更加准确,更能真实地反应中药的质量状况^[9,10]^,在中药质量控制方面有广泛的应用^[11,12]^。化学模式识别技术是一种根据物质所含化学成分信息,借助计算机处理以揭示事物内部规律的综合技术^[13]^,利用化学模式识别技术将指纹图谱中的有效信息进行综合、降维和分类分析,可以更加准确、全面、科学地对中药材进行质量评价。

本研究建立了复方金钱草颗粒的超高效液相色谱-紫外检测(UPLC-UV)定量指纹图谱,并利用超高效液相色谱-四极杆-飞行时间质谱(UPLC-Q-TOF-MS)技术对指纹图谱共有峰进行解析,结合化学模式识别技术对不同年份的复方金钱草颗粒进行质量评价,为复方金钱草颗粒的质量控制提供依据。

## 1 实验部分

### 1.1 仪器、试剂与材料

Waters Acquity I Class超高效液相色谱系统,包括四元泵、PDA检测器、自动进样器、柱温箱和Empower色谱工作站(Waters,美国); Agilent Infinity Ⅱ 1290-6545超高效液相色谱-四极杆-飞行时间质谱仪(Agilent,美国); KQ-500 DV型数控超声波清洗器(昆山市超声仪器有限公司); XSE105型十万分之一分析天平、ML204T/02型万分之一分析天平(梅特勒-托利多仪器上海有限公司); Sorvall ST8型高速离心机(赛默飞世尔苏州仪器有限公司)。

色谱级乙腈、色谱级甲酸、质谱级乙腈购自Fisher公司;超纯水由Milli-Q纯水机制备,其余试剂均为分析纯。对照品芒果苷(批号111607-201704,纯度98.1%)、毛蕊花糖苷(批号111909-201906,纯度98.0%)均购自中国食品药品检定研究院;异芒果苷(批号PS010037,纯度98.0%)、维采宁-3(批号PS020541,纯度98.0%)、异牡荆苷(批号PS001063,纯度≥98.0%)、异荭草素(批号PS001039,纯度>98.0%)、夏佛塔苷(批号PS010457,纯度>98.0%)、维采宁-2(批号PS012054,纯度>98.0%)均购自成都普思生物科技有限公司。2种规格的复方金钱草颗粒样品共36批,规格(1)为10 g/袋,规格(2)为3 g/袋,均购自广西万通制药有限公司,具体信息见表1。

**表1:** 36批复方金钱草颗粒样品信息

No.	Lot number	Size/ (g/bag)	No.	Lot number	Size/ (g/bag)
S1	180306/1	3	S19	191107/2	10
S2	180401/3	3	S20	200313/1	3
S3	181121/2	3	S21	200603/1	3
S4	180126/1	10	S22	201005/1	3
S5	180304/1	10	S23	201128/1	3
S6	180411/1	10	S24	200411/1	10
S7	180517/2	10	S25	200528/2	10
S8	180929/1	10	S26	200613/1	10
S9	181109/2	10	S27	201015/2	10
S10	190213/1	3	S28	210106/1	3
S11	190326/1	3	S29	210220/2	3
S12	190522/2	3	S30	210503/1	3
S13	190722/1	3	S31	210716/1	3
S14	190120/1	10	S32	210122/2	10
S15	190426/1	10	S33	210223/2	10
S16	190525/2	10	S34	210505/1	10
S17	190618/2	10	S35	210623/1	10
S18	190718/2	10	S36	201026/2	10

### 1.2 对照品溶液的制备

取芒果苷、异芒果苷对照品适量,加50%甲醇水溶液配制成质量浓度分别为83.2673 mg/L和65.3856 mg/L的混合对照品溶液;取毛蕊花糖苷、维采宁-3、异牡荆苷、异荭草素、夏佛塔苷及维采宁-2对照品适量,加50%甲醇水溶液配制成质量浓度分别为396、200、290、258、344及216 mg/L的混合对照品溶液。

### 1.3 供试品溶液的制备

取10袋样品,混匀,取适量,研细后取约2 g规格(1)样品或0.6 g规格(2)样品,精密称定后移至具塞锥形瓶中;加入25 mL 50%甲醇水溶液,密塞,称重,超声处理(功率300 W,频率40 kHz)10 min;将样品取出,放冷,再称定质量,用50%甲醇水溶液补足损失的质量,摇匀后离心(10000 r/min)10 min,取上清液过滤,取续滤液,即得供试品溶液。

### 1.4 仪器条件

#### 1.4.1 UPLC条件

色谱柱:Waters Acquity UPLC BEH C18(100 mm×2.1 mm, 1.7 μm), 流动相:A相为乙腈,B相为0.1%甲酸水溶液,梯度洗脱。梯度洗脱条件:0~5 min, 9%A; 5~10 min, 9%A~12%A; 10~18 min, 12%A~20%A。检测波长:350 nm;体积流量:0.4 mL/min;进样量:1 μL;柱温:35 ℃。

#### 1.4.2 LC-MS/MS条件

LC-MS/MS的色谱条件同1.4.1节;离子源:电喷雾双喷离子源(Dual AJS ESI);扫描模式:一级质谱采集(MS),自动二级质谱采集(auto MS/MS);正、负离子扫描模式;采集范围:MS *m/z* 100~1000, MS/MS *m/z* 50~1000;气帘气温度:320 ℃;鞘气温度:320 ℃;干燥气流速:8 L/min;离子化电压:+3500 V和-3000 V;毛细管出口电压:135 V;碰撞能量:20 eV和40 eV。

## 2 结果与讨论

### 2.1 指纹图谱的建立

取35批复方金钱草颗粒样品(S1~S35),分别按照1.3节中的方法制备供试品溶液,每批样品平行制备2份供试品溶液,按照1.4.1节中的方法依次进样检测,获得复方金钱草颗粒的UPLC指纹图谱(见图1)。将统一积分后的数据全部导入国家药典委员会颁布的《中药色谱特征图谱相似度评价系统软件》(2012版),以S1批样品的图谱为参照,时间窗口设置为0.1,使用平均数进行自动匹配,加以多点校正,确定12个共有色谱峰,建立了复方金钱草颗粒的UPLC对照指纹图谱(见图2)。

**图1:**
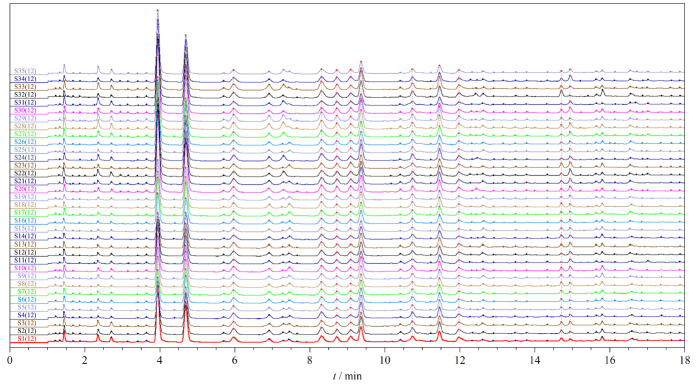
35批复方金钱草颗粒的UPLC指纹图谱

**图2:**
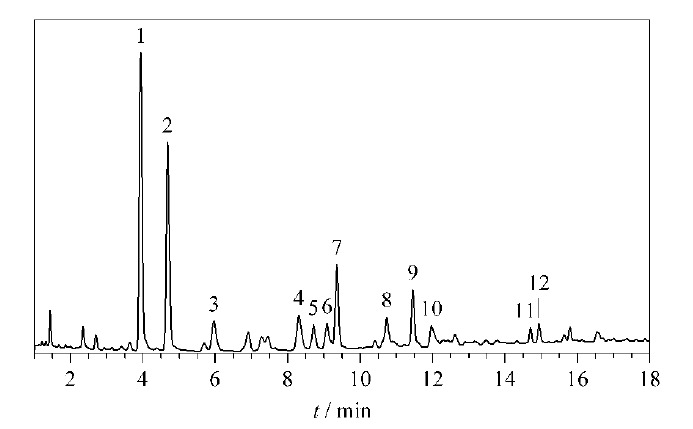
复方金钱草颗粒的UPLC对照指纹图谱

采用1.4.2节中的质谱条件进行定性分析,通过分析化合物的保留时间、一级离子质荷比以及二级碎片离子信息,结合文献、数据库及对照品比对,在复方金钱草颗粒对照指纹图谱中共指认出12个特征峰,主要为黄酮类和苯丙素类化合物。其中芒果苷、异芒果苷为光石韦中的专属成分,维采宁-2、夏佛塔苷、维采宁-3、维采宁-3的异构体为广金钱草中的专属成分,异牡荆苷为玉米须的专属成分,毛蕊花糖苷、大车前苷为车前草的专属成分(见表2),该结果为进一步研究复方金钱草颗粒的化学成分组成及阐明其药效物质基础提供了参考依据。

**表2:** 复方金钱草颗粒指纹图谱特征峰的鉴定

No.	Adduct ion	MS (m/z)	MS/MS (m/z)	Identification	Medicine
1^*^	[M+H]^+^	423.0969	405.0813, 387.0701, 369.0599, 357.0597, 327.0496, 303.0498, 273.0392, 261.0386	mangiferin^[14]^	GSW
2^*^	[M+H]^+^	423.0962	405.0809, 387.0708, 369.0599, 357.0600, 327.0495, 303.0497, 273.0390, 261.0385	isomangiferin^[15]^	GSW
3^*^	[M+H]^+^	595.1653	559.1417, 541.1321, 457.1122, 379.0803, 337.0698, 325.0694	vicenin-2^[16]^	GJQC
4	[M+H]^+^	565.1549	547.1441, 529.1330, 511.1224, 499.1219, 493.1121, 475.1218, 457.1100, 445.0879, 427.1018, 409.0911, 379.0817, 349.0682, 325.0695	vicenin 3 isomer^[16]^	GJQC
	[M+H]^+^	581.1497	563.1407, 545.1272, 527.1183, 515.1149, 497.1050, 473.1063, 461.1066, 455.0937, 443.0955	kaempferol-3-O-β-D- glucopyranosyl-7-O- β-D-apioside^[17]^	GSW
5^*^	[M+H]^+^	449.1073	431.0955, 413.0846, 395.0740, 383.0736, 353.0645, 329.0649, 299.0545	isoorientin^[16,18]^	GJQC/YMX
6	[M+H]^+^	449.1074	431.0964, 413.0858, 395.0765, 353.0643, 329.0639, 299.0536	orientin^[16,18]^	GJQC/YMX
7^*^	[M+H]^+^	565.1557	547.1433, 529.1325, 511.1233, 499.1224, 493.1118, 469.1125, 457.1117, 445.1114, 427.1022, 409.0909, 397.0912, 379.0798, 349.0692, 325.0709	schaftoside^[19]^	GJQC
8	[M+H]^+^	565.1549	547.1438, 529.1321, 511.1232, 499.1222, 493.1124, 469.1115, 457.1128, 445.1124, 427.1022, 409.0909, 397.0912, 379.0798, 349.0692, 325.0709	vicenin 3 isomer^[16]^	GJQC
9^*^	[M+H]^+^	565.1551	547.1445, 529.1333, 511.1226, 499.1223, 493.1124, 469.1120, 457.1116, 445.1128, 427.1014, 409.0913, 397.0915, 379.0811, 325.0692	vicenin 3^[16]^	GJQC
10^*^	[M+H]^+^	433.1128	415.1011, 397.0905, 379.0809, 367.0817, 337.0702, 313.0696, 283.0592	isovitexin^[6]^	YMX
11^*^	[M-H]^-^	623.1975	461.1651, 179.0349, 161.0243, 135.0449, 113.0244, 89.0244, 71.0141	acteoside^[4,20]^	CQC
12	[M-H]^-^	639.1930	477.1602, 315.1091, 161.0242, 443.0241, 71.0137	plantamajoside^[4]^	CQC

*Compared with standards and literatures; GSW: Folium Pyrrosiae Calvatae; GJQC: Desmodii Styracifolii Herba; YMX: Stigma Maydis; CQC: Plantaginis Herba.

### 2.2 指纹图谱方法学考察

精密度试验 取复方金钱草颗粒样品S36,按照1.3节中的方法制备供试品溶液,注入液相色谱仪,连续进样6次;按照1.4.1节条件进行测定,以2号峰异芒果苷为参照峰,计算共有峰相对保留时间的相对标准偏差(RSD)值为0.02%~0.13%,相对峰面积RSD值为0.25%~1.08%,结果表明仪器精密度良好。

稳定性试验 取复方金钱草颗粒样品S36,按照1.3节中的方法制备供试品溶液,分别于制备后的0、2、4、8、12、24 h注入液相色谱仪;按照1.4.1节条件进行测定,以2号峰异芒果苷为参照峰,计算共有峰的相对保留时间RSD值为0.02%~0.08%,相对峰面积RSD值为0.24%~2.26%,结果表明供试品溶液在24 h内稳定。

重复性试验 取复方金钱草颗粒样品S36,精密称定6份,按照1.3节中的方法平行制备6份供试品溶液;按照1.4.1节条件进行测定,以2号峰异芒果苷为参照峰,计算共有峰相对保留时间的RSD值为0.02%~0.11%,相对峰面积的RSD值为0.51%~1.29%,结果表明本方法重复性良好。

### 2.3 指纹图谱评价

#### 2.3.1 相似度评价

将得到的35批复方金钱草颗粒指纹图谱导入《中药色谱指纹图谱相似度评价系统》(2012版),进行色谱峰匹配,计算不同批次样品与对照指纹图谱的相似度,结果见表3。结果显示样品的相似度均≥0.952,表明35批样品的指纹图谱相似度较高,不同批次样品的质量相对稳定。

**表3:** 35批复方金钱草颗粒样品的相似度

No.	Similarity	No.	Similarity
S1	0.999	S19	0.997
S2	0.995	S20	0.996
S3	0.999	S21	0.996
S4	0.954	S22	0.993
S5	0.952	S23	0.987
S6	0.966	S24	0.999
S7	0.968	S25	0.990
S8	0.989	S26	0.993
S9	0.990	S27	0.999
S10	0.989	S28	0.996
S11	0.998	S29	0.996
S12	0.996	S30	0.995
S13	0.993	S31	0.989
S14	0.981	S32	0.998
S15	0.972	S33	0.998
S16	0.992	S34	0.996
S17	0.995	S35	0.992
S18	0.991		

#### 2.3.2 聚类分析(HCA)

分别将35批复方金钱草颗粒样品的12个共有峰进行峰面积归一化计算,将归一化后的数据导入Origin Pro软件中进行层次聚类分析,聚类图见图3。结果显示35批样品可以分为2类,第1类为S1~S19,第2类为S20~S35,即2018年与2019年的样品聚为一类,2020年与2021年的样品聚为一类,表明HCA能明显区分2018年、2019年与2020年、2021年生产的样品。

**图3:**
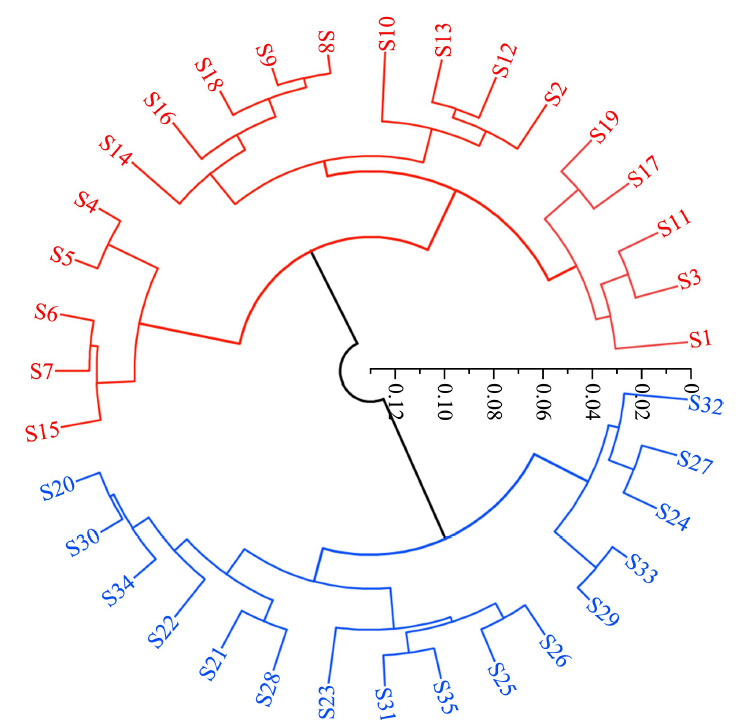
35批复方金钱草颗粒的聚类分析

#### 2.3.3 主成分分析(PCA)

将35批复方金钱草颗粒样品归一化峰面积导入SIMCA 14.0软件中,进行无监督的主成分分析^[21]^, PCA得分见图4。从图4中可以看到,PCA结果与HCA结果一致,即2018年、2019年的样品与2020年、2021年的样品有明显的分类趋势。但由于数据点较为零散,考虑进一步采用有监督的模型进行分析。

**图4:**
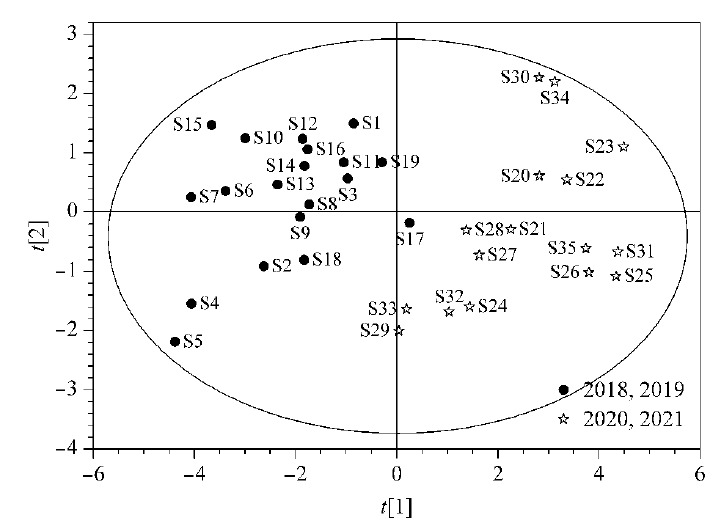
复方金钱草样品PCA分析得分图

#### 2.3.4 正交偏最小二乘判别分析(OPLS-DA)

为了更好地分析2018年、2019年与2020年、2021年样品的组间差异,在PCA分组的基础上,进行有监督的正交偏最小二乘判别分析,获得相应模型。在该模型下*R*^2^*X*值为0.922、*R*^2^*Y*值为0.563、*Q*^2^值为0.518,三者均大于0.5,表明该模型具有较好的预测能力;进一步采用200次响应排序检验验证模型是否存在过拟合现象。结果如图5所示,所有通过随机排序计算得到的*R*^2^和*Q*^2^值均小于原始值,且*Q*^2^回归直线与Y轴间存在负截距,说明该模型是有效的且不存在过度拟合现象^[22]^,可用于复方金钱草颗粒差异标志物的筛选。OPLS-DA结果显示,35批样品被分为2类,如图6所示,2018年、2019年的样品聚为一类,但样品点分布较为分散,表明2018年、2019年生产的样品质量差异较大;而2020年、2021年的样品聚为一类,样品点分布集中,表明2020年、2021年生产的样品中组内质量更均一稳定。结果表明OPLS-DA可对2018年、2019年与2020年、2021年生产的样品进行很好的聚类区分。

**图5:**
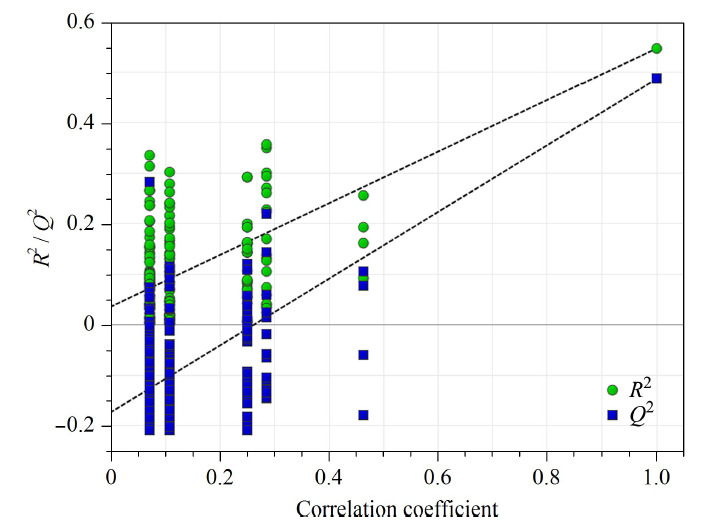
OPLS-DA模型的200次响应排序检验

**图6:**
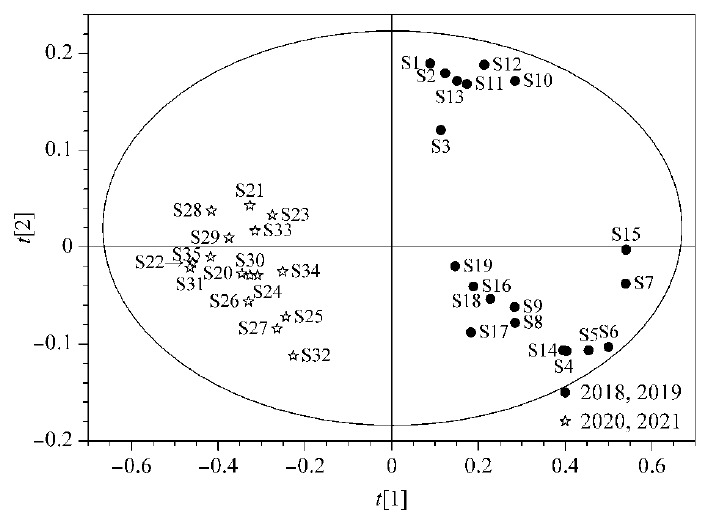
35批复方金钱草颗粒的OPLS-DA图

为进一步筛选出导致2018年、2019年与2020年、2021年样品产生差异的成分,根据模型中变量投影重要度排序(VIP)预测值筛选出VIP值大于1的色谱峰为差异标志物。如图7所示,芒果苷、异芒果苷的VIP值均大于1,说明芒果苷和异芒果苷可能是区分2018年、2019年与2020年、2021年复方金钱草颗粒样品的差异性质量标志物。

**图7:**
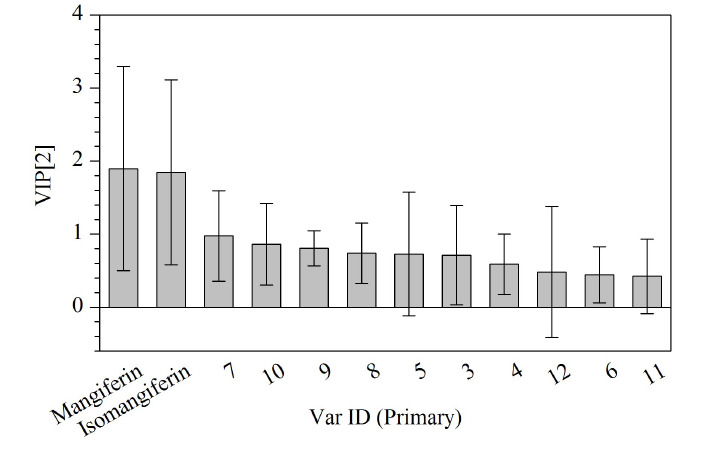
复方金钱草颗粒的OPLS-DA的VIP值

### 2.4 芒果苷和异芒果苷含量测定

文献研究表明芒果苷和异芒果苷均具有抗炎、免疫、抗菌等作用^[23,24]^,二者与复方金钱草颗粒的药理功效密切相关,且通过化学模式识别技术筛选出了两个质量差异标志物,即芒果苷和异芒果苷,故对二者进行含量测定。

#### 2.4.1 线性关系考察

取芒果苷、异芒果苷对照品适量,加50%甲醇水溶液配制成7个质量浓度的系列混合对照品溶液,其中芒果苷的质量浓度为133.2276、106.5821、79.9366、53.2911、26.6455、13.3228、5.3291 mg/L;异芒果苷的质量浓度为104.6170、83.6936、62.7702、41.8468、20.9234、10.4617、4.1847 mg/L。分别精密吸取1 μL对照品溶液,按1.4.1节条件进样测定,记录色谱图。以对照品质量浓度为横坐标(*x*),相应的峰面积积分值为纵坐标(*y*),分别绘制标准曲线,得线性回归方程、相关系数(*R*^2^)和线性范围,结果见表4。芒果苷和异芒果苷在一定浓度范围内呈良好的线性关系,计算回归方程的截距误差,结果均小于2%,表明线性关系准确可靠。

**表4:** 芒果苷和异芒果苷的线性回归方程、相关系数、线性范围及截距误差

Compound	Linear regression equation	R^2^	Linear range/(mg/L)	Intercept error/%
Mangiferin	y=3103.7x-4181.5	0.9998	5.3291-133.2276	1.73
Isomangiferin	y=2776.1x-3129.6	0.9998	4.1847-104.6170	1.84

*y*: peak area; *x*: mass concentration, mg/L.

#### 2.4.2 精密度试验

精密吸取复方金钱草颗粒供试品(S36)溶液,连续进样6次,记录芒果苷和异芒果苷的色谱峰面积,计算芒果苷和异芒果苷含量的RSD值分别为0.64%和0.14%,表明仪器精密度良好。

#### 2.4.3 重复性试验

精密称取复方金钱草颗粒样品(S36),按1.3节中的方法制备6份供试品溶液,按1.4.1节条件进样测定,记录芒果苷和异芒果苷的色谱峰面积,计算芒果苷和异芒果苷含量的RSD值分别为0.52%和0.28%,表明该方法重复性良好。

#### 2.4.4 稳定性试验

精密吸取复方金钱草颗粒供试品(S36)溶液,按1.4.1节条件分别于制备后的0、2、4、8、12、24 h进样,记录芒果苷和异芒果苷的色谱峰面积,计算芒果苷和异芒果苷含量的RSD值分别为0.71%和0.12%,表明供试品溶液在24 h内稳定性良好。

#### 2.4.5 加标回收率试验

精密称取已测定的同一批次复方金钱草颗粒(S36)适量,按其中芒果苷、异芒果苷含量约50%、100%、150%浓度水平准确加入已知量的2种对照品混合溶液,按1.3节中的方法制成低、中、高浓度各3份的供试品溶液,记录芒果苷和异芒果苷的色谱峰面积,计算芒果苷和异芒果苷的平均加标回收率分别为101.7%~105.6%和103.4%~105.5%,均在《中国药典》(2020版)规定的限度范围(90%~108%)内^[25]^,RSD值分别为0.63%~1.43%和0.60%~1.18%,表明方法的准确性良好。

#### 2.4.6 检出限和定量限

取1.2节中芒果苷和异芒果苷的混合对照品溶液,分别加50%甲醇水溶液进行逐级稀释,按1.4.1节条件进行测定,以3倍信噪比(*S/N*=3)确定检出限,10倍信噪比(*S/N*=10)确定定量限^[25]^。结果显示芒果苷的检出限为0.2667 mg/L,定量限为0.8889 mg/L;异芒果苷的检出限为0.3394 mg/L,定量限为1.1310 mg/L。

#### 2.4.7 样品含量测定

将35批复方金钱草颗粒按1.3节中的方法制备供试品溶液,按1.4.1节条件进行测定,计算样品中芒果苷和异芒果苷的含量,35批样品中芒果苷和异芒果苷含量的累积加和图见图8。

**图8:**
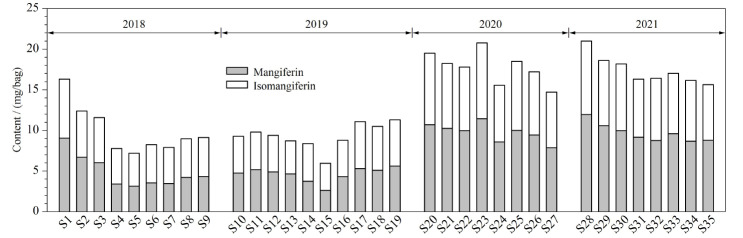
35批复方金钱草颗粒指标成分累积加和图

由图8可直观看出,不同批次样品中的成分含量存在一定差异,2020年、2021年的样品较2018年、2019年的样品含量更高;计算2018年、2019年样品含量的波动范围,芒果苷为55%~192%,异芒果苷为68%~149%;而计算2020年、2021年样品含量的波动范围,芒果苷为81%~123%,异芒果苷为87%~119%。上述结果显示2020年、2021年与2018年、2019年的样品相比,芒果苷与异芒果苷含量更高且波动范围更小,表明2020年、2021年的样品质量较好且相对稳定。为保证复方金钱草颗粒的质量,在今后的药品生产中也应重点关注这两个成分的含量变化。

## 3 结论

本文首先建立了一种复方金钱草颗粒的UPLC-UV指纹图谱,指认了其中12个共有峰,并结合化学模式识别技术筛选出了两个质量差异标志物芒果苷和异芒果苷,并对二者进行了含量测定。结果表明,该方法可对复方金钱草颗粒实现快速的定性定量分析,利用指纹图谱进行化学模式识别,再结合含量测定可对不同年份的复方金钱草颗粒进行质量评价,从而有效、准确地对复方金钱草颗粒进行全面的综合评价,为复方金钱草颗粒的质量控制提供科学的方法与依据。
